# Genotoxicity and acute toxicity evaluation of the three amino acid additives with *Corynebacterium glutamicum* biomass

**DOI:** 10.1016/j.toxrep.2020.01.013

**Published:** 2020-01-27

**Authors:** Ki-Young Kang, Min-Sub Kim, Min-Seung Lee, Jeong-Ja Oh, Seulgi An, Dhanbee Park, In Kyoung Heo, Hyun-Kul Lee, Si-Whan Song, Sun-Don Kim

**Affiliations:** a Safety Evaluation Center, Nonclinical Research Institute, ChemOn Inc., 240, Nampyeong-ro, Yangji-myeon, Cheoin-gu, Yongin-si, Gyeonggi-do, 17162, Republic of Korea; b BIO) Registration Team, CJ Cheiljedang Center, 330, Dongho-ro, Jung-gu, Seoul, 04560, Republic of Korea; c CJ Research Institute of Biotechnology, 55, Gwanggyo-ro 42beon-gil, Yeongtong-Gu, Suwon-si, Gyeonggi-do, 16495, Republic of Korea

**Keywords:** Ames test, Chromosomal aberration test, Acute oral toxicity, Amino acid additives, *Corynebacterium glutamicum*

## Abstract

l-threonine, l-tryptophan and l-valine play a fundamental role in animal and human nutrition as essential amino acids required for normal growth. In addition, each amino acid is codified as a generally recognized as safe (GRAS) amino acid for the use in animal feed additives and presents no exposure risk from animal to humans consuming tissues or products from the target animal. Taking into account the important role of mutagenicity and genotoxicity in the risk of the three amino acid additives (l-threonine, l-tryptophan, and l-valine) fermentation products and other unknown impurities and derivatives from *Corynebacterium glutamicum* (*C. glutamicum*), the safety evaluation of these amino acid additives is not performed. Therefore, the purpose of this study is to evaluate toxicological effects, including Ames test, an *in vitro* mammalian chromosomal aberration test and an acute oral animal toxicity of the three amino acid additives in accordance with the Organisation for Economic Co-operation and Development (OECD) guidelines and the principles of Good Laboratory Practice (GLP). As a result, these amino acid additives were classified as non-mutagenic and non-clastogenic, and did not induce any toxicity in acute oral toxicity test. Collectively, these results suggest that the three amino acid additives are safe with no adverse effects, and able to be applied as an ingredient or other biological uses.

## Highlights

• The three amino acid additives with C. *glutamicum* were found to be non-mutagenic and non-toxic.
• Genotoxicity is not observed in Ames assay and mammalian cytogenentic assay of the three amino acid additives.
• No clinical signs, mortalities, abnormal necropsy findings were observed in acute oral toxicity in both Sprague-Dawley and Wistar rats.

## 1 Introduction

Dietary ingestion of amino acids in domestic animals has been widely investigated and the important role of amino acids in modulation of metabolism, growth, reproduction and milk production was unveiled [1]. Accordingly, the addition of amino acids in animal feed is a reasonable option for livestock and the animal breeding industry. For instance, l-tryptophan is one of the essential amino acids in mammals which cannot be synthesized. Diverse roles of L-tryptophan were identified such as food intake enhancer, growth contributor, and immune response modulator [2]. l-threonine is one of the first-limiting amino acids in low protein diet of animals and supplementation of threonine was reported to improve lipid metabolism in poultry and to increase whole-body protein synthesis in weanling colts [3,4]. Deprivation of branched chain amino acid l-valine was known to remarkably decrease insulin sensitivity of mice and impair immune system of young grass carp [5,6]. On the contrary, optimal dietary supplementation of valine increases body weight gain of Pekin duck [7].

*Corynebacterium glutamicum* (*C. glutamicum*) is a gram-positive and non-pathogenic bacterium, which occurs naturally in soil and is generally recognized as safe (GRAS) organism [8]. *C. glutamicum* was listed on the Qualified Presumption of Safety (QPS) list of European Food Safety Authority (EFSA) and the concerns for the safety of *C. glutamicum* when used for biotechnological process are rarely considered [9]. Recently, this microorganism has shown remarkable potential of biological production of various nutrients including organic acids, vitamins, and amino acids [[10], [11], [12]]. Therefore, the importance of *C. glutamicum* as a role of industrial workhorse in the fields of medicine, biotechnology, and agriculture has been more emphasized.

The safety of *C. glutamicum* and the three amino acid additives produced through the biotechnological process in *C. glutamicum* has been evaluated for decades. However, the safety of amino acid additives such as l-threonine, l-tryptophan, and l-valine fermentation products produced by *C. glutamicum* and other unknown impurities and derivatives from *C. glutamicum* had not been previously investigated. Therefore, in this study, the genotoxicity of amino acid additives and *C. glutamicum’*s biomass was evaluated by using a bacterial reverse mutation test and an *in vitro* mammalian chromosomal aberration test. Furthermore, a single oral dose study was performed to evaluate the acute toxicity.

## 2 Materials and methods

### 2.1 Chemicals

Test compounds of amino acids, l-threonine, l-valine, and l-tryptophan fermentation products were obtained from CJ CheilJedang Corporation (Seoul, Republic of Korea) and their compositions are shown in Table 1. Benzo[*a*]pyrene (B[a]P), 4-Nitroquinoline-1-oxide (4NQO), Mitomycin C (MMC), Cyclophosphamide monohydrate (CPA), 2-aminoanthracene (2-AA), sodium azide (SA), 2-Nitrofluorene (2-NF), Dimethyl sulfoxide (DMSO) and Acridine Mutagen ICR 191 (ICR-191) were acquired from Sigma-Aldrich (St. Louis, MO, USA). Fetal bovine serum (FBS), Penicillin-Streptomycin, GlutaMax-I, Minimum Essential Medium (MEM) and Phosphate-Buffered Saline were obtained from Invitrogen (Gibco, NY, USA).

**Table 1:** Compositions of test compounds.

Test compound	Components	Compositional average (%)
L-threonine fermentation product	L-threonine	77.94
	Total, other than l-threonine (hydrolyzed amino acids, free amino acids, organic acids, ammonium, sugars, *etc*.)	20.65
	Moisture	1.99
	Unknown impurities and derivatives of *Corynebacterium glutamicum*	trace
L-valine fermentation product	L-valine	72.66
	Total, other than l-valine (hydrolyzed amino acids, free amino acids, organic acids, ammonium, sugars, *etc*.)	25.75
	Moisture	1.19
	Unknown impurities and derivatives of *Corynebacterium glutamicum*	trace
L-tryptophan fermentation product	L-tryptophan	63.09
	Total, other than l-tryptophan (hydrolyzed amino acids, free amino acids, organic acids, ammonium, sugars, *etc*.)	33.64
	Moisture	2.56
	Unknown impurities and derivatives of *Corynebacterium glutamicum*	trace

### 2.2 Animals

The Specific pathogen free (SPF) Sprague-Dawley (Crl:CS(SD)) and Wistar (RccHan™:WST) rats for acute oral toxicity were obtained from Orient Bio Inc. (Seongnam, Republic of Korea) and Envigo RMS (UK) limited (Oxon, UK), respectively. The animals were maintained under constant environmental conditions (temperature, 23 ± 3 °C; humidity, 55 ± 15%; ventilation, 10–20 air changes/hour, and luminous intensity, 150–300 Lux in the barriered experimental animal facility at Chemon Inc., Nonclinical Research Institute accredited by AAALAC International from March 02, 2010 #001333 in accordance with Guide for the Care and Use of Laboratory Animals, 8th edition (2010) or Envigo Research Limited on animal welfare and the requirement of the United Kingdom’s Animals (Scientific Procedures) Act 1986 Amendment Regulation 2012 [13]. Food and water were provided, *ad libitum*, with 12 h light: 12 h dark cycle. All procedures and protocols were reviewed and approved by Institutional Animal Care and Use Committee (IACUC) of Chemon Inc and Envigo Research Limited and performed in accordance with the guideline published by the Organisation for Economic Co-operation and Development (OECD) as well as the UK Home Office Guidance documents on Regulatory Toxicology and Safety Evaluation Studies or Good Laboratory Practice (GLP) regulations for Nonclinical Laboratory Studies of the Ministery of Food Drug Safety [14,15].

### 2.3 Bacterial strains and culture conditions

Five *Salmonella typhimurium* (*S. typhimurium*) histidine-auxotrophic strains TA98, TA100, TA102, TA1535, and TA1537 and one *Escherichia coli* (*E. coli*) tryptophan-auxotrophic strain WP2 uvrA were obtained from Molecular Toxicology Inc. (NC, USA) and used for the Ames test. The broth for the mutagenicity assay was 2.5 % Oxoid Nutrient Broth No. 2. The minimal glucose agar was Vogel-Bonner medium E supplemented with 1.5 % Bacto Agar and 2 % glucose. The minimal glucose agar for the WP2 uvrA strain was supplemented with additional 0.25 mL/L of 0.1 % l-tryptophan. Top agar for selection of revertants was prepared with 0.6 % Bacto agar and 0.5 % NaCl. The top agar for Salmonella strains was supplemented with 10 mL of 0.5 mM histidine/biotin solution per 100 mL. Cultivated Chinese hamster lung fibroblast cells (CHL/IU, #CRL-1935) were obtained from the American Type Culture Collection (VA, USA). Briefly, cells were cultured in MEM medium (D-glucose, 1000 mg/mL; CaCl_2_, 1.81 mM; MgSO_4_, 0.81 mM; KCl, 5.33 mM; NaCl, 117.24 mM; NaHCO_3_, 26.19 mM; l-Glutamic acid, 0.10 mM; Glycine, 0.10 mM; l-threonine, 0.40 mM; l-tryptophan, 0.05 mM; l-valine, 0.39 mM) supplemented with Glutamax-1 supplement, penicillin-streptomycin, and 10 % fetal bovine serum. Cultures were incubated in a humidified incubator at 37 ± 1 °C with 5 % CO_2_ in air and sub-cultured every 2–3 days using 0.1 % trypsin solution.

### 2.4 Bacterial reverse mutation test (Ames test)

Under GLP regulation, the Ames test was performed (referencing the OECD Guideline 471 [16], Maron and Ames [17], Vogel and Bonner [18], Green and Muriel [19], and Kilbey et al. [20]) with minor modifications as follows: five histidine auxotroph strains of *S. typhimurium* TA100, TA1535, TA98, TA1537, TA102, and a tryptophan auxotroph strain of *E. coli* WP2 *uvr*A were obtained from Molecular Toxicology Inc. (NC, USA) and were cultured in maintenance or growth media in accordance with the provider’s instructions. The mutagenic activity of each test compound was assessed in the absence and presence of external metabolic activation system from rat livers (S9 fraction) using the direct plate incorporation method. For the plating assay, 0.5 mL of S9 mix (or sodium-phosphate buffer, pH 7.4 for non-activation plates), 0.1 mL of bacterial culture (containing approximately 10^8^ viable cells), and 0.1 mL of test compound were mixed with 2.0 mL of overlay agar. The contents of each tube were mixed and poured over the surface of a minimal agar plate. The overlay agar was allowed to solidify before incubation. After the top was solidified, plates were inverted and incubated at 37 ± 2 °C for 50 ± 2 h and the revertant colonies were counted with unaided eye. The growing concentrations of each test compound were selected based on the results of a range-finding test conducted on the test compound using the five or six test strains in both the presence and absence of S9 fraction. One experiment was conducted with five growing concentrations (50, 150, 500, 1500, and 5000 μg/plate for the l-valine and l-tryptophan fermentation products). The other experiment was conducted with six growing concentrations (12, 37, 111, 333, 1000, and 3000 μg/plate for the l-threonine fermentation product). Negative control (distilled sterile water), a solvent control (DMSO) and positive controls for each strain in accordance with the presence or absence of S9 fraction were included. Positive controls in the absence of S9 fraction were as follows: SA (0.5 μg/plate) for TA100 and TA1535; 2-NF (2 μg/plate) for TA98; Acridine Mutagen ICR 191 (0.5 μg/plate) for TA1537; 4NQO (0.5 μg/plate) for WP2 uvrA; and MMC (0.5 μg/plate) for TA102. In addition, positive controls in the presence of S9 fraction were as follows: 2-AA (1 μg/plate, TA100 and TA1537; 2 μg/plate, TA1535; 6 μg/plate, TA102 and WP2 uvrA) for TA100, TA1535, TA1537, and WP2 uvrA and B[a]P (1 μg/plate) for TA98. At least 3 independent experiments were performed using triplicate plates for each concentration. Results are expressed as number of revertant colonies, and the increase factor was calculated by dividing the number of colony of the treated plates with the number of colony of the negative control plates.

### 2.5 *In vitro* mammalian chromosomal aberration test

The chromosomal aberration test is designed to evaluate the mutagenic potential to induce structural and/or numerical chromosomal aberrations in cultured CHL cells*. In vitro* chromosomal aberration test was performed according to OECD guidelines 473 [21], Ishidate et al. [22] and Dean and Danford [23] under GLP regulation. Structural abnormalities (g, gap of chromatid or chromosome; ctb, break of chromatid; cte, exchange of chromatid; csb, break of chromosome; cse, exchange of chromosome) and polyploidy as chromosomal aberrations were evaluated in cultured CHL cells in the presence and absence of exogenous metabolic activation system, consisting of the cofactor-supplemented post-mitochondrial fraction (S9) of liver homogenates from rats treated with Aroclor 1254 [24]. CHL/IU cells were cultured for 3 days from an aliquot of 5 × 10^4^ cells per flask (culture surface, 25 cm^2^) [25]. Short-term (6–8 hours) and continuous (24 h) treatments were performed as follows: concentrations of l-threonine fermentation product were 500, 1000, and 2000 μg/mL for 6 + S, 6-S, and 24-S; concentrations of l-valine fermentation product were 250, 500, 800, and 1000 μg/mL for 6 + S, 6-S and 24-S; concentrations of l-tryptophan fermentation product were 0, 87.5, 125, 175, 250, 310, 350, and 500 μg/mL for 6 + S, 6-S and 24-S. Approximately 22 h after treatment, 50 μL of colchicine solution was added to each culture (final concentration of 1 μM) and incubated for 2 h for mitotic arrest. The mitotic cells were detached by gentle shaking. The medium containing mitotic cells were centrifuged, and the cell pellets were resuspended in 75 mM potassium chloride solution for hypotonic treatment. Then cells were fixed with fixative (methanol:glacial acetic acid = 3:1 v/v) three times and slides were prepared by the air-drying method. Slides were stained with 5 % Giemsa solution. Two slides were prepared for each culture. One hundred and fifty metaphases per flask (300 metaphases per concentration) were evaluated for chromosome aberrations. The results were expressed as frequency (%) of metaphases with structural or numerical aberrations per 300 metaphases. Relative increase in cell count (RICC) was used as an indicator of concurrent cytotoxicity to determine the high concentration. With the cell counts, RICC was calculated as follows and used as an indicator of concurrent cytotoxicity.

$$RICC\;=\;\;\frac{\left(Cell\;count\;of\;\;treated\;\;flask\;-\;Initial\;cell\;count\right)}{\left(Cell\;count\;of\;control\;flask\;-\;Initial\;cell\;count\right)}\;\times 100\;(\%)$$

The selection of maximum concentration was based on cytotoxicity, and the highest concentration aimed to achieve 55 ± 5% cytotoxicity. All concentrations of test compounds as well as the concurrent negative (vehicle) and the positive control (benzo[*a*]pyrene (B[a]P) and 4-nitroquinoline-1-oxide (4NQO) for l-valine and l-tryptophan fermentation products; Mitomycin C (MMC) and Cyclophosphamide monohydrate (CPA) for l-threonine fermentation product), were evaluated using duplicate cultures per concentration.

### 2.6 Acute oral toxic class method study

The present study was performed to assess the toxicity of l-threonine fermentation product in Wistar rats and l-valine and l-tryptophan fermentation products in Sprague-Dawley rats following a single oral administration acute toxic method according to OECD guideline 420 [26]. Stepwise procedure with two fixed doses (a sighting test in one female at 300 mg/kg b.w. and in one female at 2000 mg/kg b.w., and further main test in four females at 2000 mg/kg b.w.) was performed. In two fixed doses, six females were treated with l-threonine, l-valine, and l-tryptophan fermentation products by oral gavage administration. Before dosing, animals were fasted overnight and then the test compound was directly administered into stomach using a syringe tube with a feeding needle. Food was given in 3 or 4 h after administration. All animals were observed for mortality and clinical signs every hour for 6 h after dosing during the first 24 h and then once daily for a total of 14 days. Body weights were recorded on day 1(prior to the administration), 2, 4, 8, and 15 after dosing of l-valine and l-tryptophan fermentation products, while they were recorded on day 1(prior to the administration), 8 and 15 after dosing of l-threonine fermentation product. At study termination, all animals were euthanized by CO_2_ inhalation and their organs were then collected for macroscopic necropsy examination.

### 2.7 Statistical analysis

Statistical analysis was performed using SPSS Statistics version 22. The level of significance was taken as *P* ≤ 0.05. Fisher’s exact test was used to compare the frequencies of aberrant metaphase between the negative control and treated groups for the chromosomal aberration test. Statistical analysis for acute oral toxicity was not performed as number of animals was insufficient for analysis.

## 3 Results

### 3.1 Bacterial reverse mutation test (Ames test)

Mutagenicity tests were performed to evaluate the test compound’s potential to induce reverse mutation in the five histidine auxotroph strains of *S. typhimurium* TA100, TA102, TA1535, TA98, TA1537 and a tryptophan auxotroph strain of *E. coli* WP2 uvrA in the presence and absence of exogenous metabolic activation system.

As shown in Table 2, Table 3, l-threonine and l-valine fermentation products showed no substantial increases in numbers of revertants per plate at concentration levels (12, 37, 111, 333, 1000, and 3000 μg/plate for l-threonine fermentation product; 50, 150, 500, 1500, and 5000 μg/plate for l-valine and l-tryptophan fermentation products) in TA100, TA1535, TA98, TA1537 and WP2 uvrA in either the presence or absence of metabolic activation of S9 mix. Also, l-tryptophan fermentation product showed no substantial increases in numbers of revertants per plate in TA100, TA102, TA1535, TA98 and TA1537 strains at concentration levels (50, 150, 500, 1500, and 5000 μg/plate) in either the presence or absence of metabolic activation of S9 mix (Table 4). However, in WP2 uvrA both in the presence and absence of metabolic activation system in Table 4, l-tryptophan induced significant increases (*P* ≤ 0.05) of the revertants per plate in WP2 uvrA. In addition, the mean revertant of the positive control for each test strain exhibited a clear increase over the mean revertant of the negative control for that strain. There was no indication of mutagenicity at all concentrations tested.

**Table 2:** Reverse mutagenicity assay of l-threonine fermentation product.

L-threonine fermentation product																																							
Conc.			Colonies/plate [factor] (With S9 mix) ^a)^																																				
(μg/plate)			*S. typhimurium*									*S. typhimurium*								*S. typhimurium*							*S. typhimurium*						*E. coli*						
			TA 100									TA1535								TA98							TA1537						WP2 *uvr*A						
0			119		±	6						10		±		1				30		±	2				14	±	1				26		±	4			
12			111		±	9		[		0.9	]	9		±		2	[	0.9	]	28		±	3	[	0.9	]	17	±	1	[	1.2	]	23		±	3	[	0.9	]
37			105		±	5		[		0.9	]	8		±		4	[	0.8	]	30		±	3	[	1	]	15	±	4	[	1.1	]	25		±	3	[	0.9	]
111			122		±	12		[		1	]	12		±		2	[	1.2	]	30		±	2	[	1	]	17	±	2	[	1.2	]	29		±	2	[	1.1	]
333			112		±	12		[		0.9	]	13		±		2	[	1.3	]	31		±	3	[	1	]	13	±	4	[	1	]	20		±	3	[	0.8	]
1000			118		±	13		[		1	]	12		±		1	[	1.2	]	34		±	5	[	1.1	]	12	±	3	[	0.9	]	22		±	1	[	0.9	]
3000	TP		–		±	–		[		–	]	–		±		–	[	–	]	–		±	–	[	–	]	–	±	–	[	–	]	–		±	–	[	–	]
Positive Control			1312		±	153			[	11.1	]	210		±		5	[	21	]	258		±	51	[	8.6	]	193	±	8	[	14.1	]	83		±	1	[	3.2	]
Colonies/plate [factor] (Without S9 mix) ^a)^																																							
0				118	±	9							10	±		2				24		±	4				11	±	1				20		±	4			
12				141	±	18		[		1.2	]		9	±		1	[	0.9	]	28		±	2	[	1.2	]	9	±	3	[	0.8	]	22		±	4	[	1.1	]
37				130	±	16		[		1.1	]		11	±		1	[	1.1	]	29		±	4	[	1.2	]	8	±	1	[	0.8	]	21		±	2	[	1.1	]
111				111	±	6		[		0.9	]		11	±		1	[	1.1	]	27		±	4	[	1.1	]	10	±	1	[	1	]	23		±	4	[	1.2	]
333				101	±	6		[		0.9	]		11	±		2	[	1.1	]	27		±	5	[	1.1	]	10	±	3	[	0.9	]	26		±	3	[	1.3	]
1000				105	±	8		[		0.9	]		10	±		3	[	1	]	29		±	4	[	1.2	]	8	±	1	[	0.8	]	24		±	2	[	1.2	]
3000	TP			–	±	–		[		–	]			±			[	–	]	–		±	–	[	–	]	–	±	–	[	–	]	–		±	–	[	–	]
Positive control				400	±	35		[		3.4	]		433	±		52	[	43.3	]	257		±	24	[	10.7	]	183	±	9	[	17.2	]	182		±	10	[	9.2	]
Strain (With S9 mix)							Positive control								Dose (μg/plate)						Strain (Without S9 mix)							Positive control						Dose (μg/plate)					
TA 100							2-AA								1						TA 100							SA						0.5					
TA1535							2-AA								2						TA1535							SA						0.5					
TA98							B[a]P								1						TA98							2-NF						2					
TA1537							2-AA								1						TA1537							ICR-191						0.5					
WP2 *uvr*A							2-AA								6						WP2 *uvr*A							4NQO						0.5					

a) Three plates/dose were used. No. of colonies of treated plate/No. of colonies of negative control plate Abbreviations: T, Turbidity in the treatment mixture; P, Precipitation in the treatment mixture; B[a]P, Benzo[*a*]pyrene; 4NQO, 4-Nitroquinoline-1-oxide; SA, sodium azide; 2-NF, 2-Nitrofluorene; ICR-191, Acridine Mutagen ICR 191; 2-AA, 2-aminoanthracene.

**Table 3:** Reverse mutagenicity assay of l-valine fermentation product.

L-valine fermentation product																																							
Conc.			Colonies/plate [factor] (With S9 mix) ^a)^																																				
(μg/plate)			*S. typhimurium*									*S. typhimurium*								*S. typhimurium*							*S. typhimurium*						*E. coli*						
			TA 100									TA1535								TA98							TA1537						WP2 *uvr*A						
0			166		±	3						23		±		2				22		±	1				14	±	1				37		±	2			
50			177		±	3		[		1.1	]	22		±		2	[	1	]	24		±	1	[	1.1	]	11	±	1	[	0.8	]	37		±	2	[	1	]
150			174		±	2		[		1	]	26		±		1	[	1.2	]	24		±	2	[	1.1	]	15	±	1	[	1.1	]	38		±	1	[	1	]
500			182		±	2		[		1.1	]	17		±		1	[	0.8	]	21		±	1	[	1	]	11	±	2	[	0.8	]	37		±	2	[	1	]
1500	T		173		±	2		[		1	]	19		±		1	[	0.9	]	21		±	2	[	1	]	12	±	2	[	0.9	]	39		±	1	[	1	]
5000	T		179		±	4		[		1.1	]	22		±		1	[	1	]	25		±	2	[	1.1	]	15	±	1	[	1.1	]	37		±	2	[	1	]
Positive control			1720		±	133			[	10.4	]	155		±		16	[	6.9	]	176		±	8	[	7.9	]	213	±	16	[	15.6	]	122		±	7	[	3.3	]
Colonies/plate [factor] (Without S9 mix) ^a)^																																							
0				170	±	2							21	±		1				21		±	1				12	±	1				39		±	3			
50				164	±	3		[		1	]		21	±		0	[	1	]	21		±	1	[	1	]	11	±	2	[	0.9	]	38		±	1	[	1	]
150				184	±	4		[		1.1	]		23	±		2	[	1.1	]	16		±	1	[	0.8	]	9	±	2	[	0.8	]	37		±	1	[	0.9	]
500				181	±	2		[		1.1	]		22	±		1	[	1.1	]	18		±	2	[	0.9	]	13	±	2	[	1.1	]	37		±	1	[	0.9	]
1500	T			172	±	4		[		1	]		23	±		2	[	1.1	]	17		±	1	[	0.8	]	10	±	1	[	0.8	]	32		±	2	[	0.8	]
5000	T			168	±	3		[		1	]		25	±		1	[	1.2	]	22		±	1	[	1	]	13	±	1	[	1.1	]	42		±	1	[	1.1	]
Positive control				304	±	39		[		1.8	]		265	±		21	[	12.8	]	194		±	3	[	9.2	]	188	±	6	[	15.3	]	142		±	17	[	3.6	]
Strain (With S9 mix)							Positive control								Dose (μg/plate)						Strain (Without S9 mix)							Positive control						Dose (μg/plate)					
TA 100							2-AA								1						TA 100							SA						0.5					
TA1535							2-AA								2						TA1535							SA						0.5					
TA98							B[a]P								1						TA98							2-NF						2					
TA1537							2-AA								1						TA1537							ICR-191						0.5					
WP2 *uvr*A							2-AA								6						WP2 *uvr*A							4NQO						0.5					

a) Three plates/dose were used. No. of colonies of treated plate/No. of colonies of negative control plate Abbreviations: T, Turbidity in the treatment mixture; B[a]P, Benzo[*a*]pyrene; 4NQO, 4-Nitroquinoline-1-oxide; 2-AA, 2-aminoanthracene; SA, sodium azide; 2-NF, 2-Nitrofluorene; ICR-191, Acridine Mutagen ICR 191.

**Table 4:** Reverse mutagenicity assay of l-tryptophan fermentation product.

L-tryptophan fermentation product																																														
Conc.						Colonies/plate [factor] (With S9 mix) ^a)^																																								
(μg/plate)						*S. typhimurium*									*S. typhimurium*													*S. typhimurium*									*S. typhimurium*									
						TA 100									TA1535													TA98									TA1537									
0						183	±			6					22			±			1							24	±	1							11		±			1				
50						186	±			4	[	1	]		21			±			1		[	1		]		25	±	0	[		1	]			10		±			1		[	0.9	]
150						182	±			1	[	1	]		23			±			2		[	1		]		24	±	2	[		1	]			10		±			1		[	0.9	]
500						186	±			5	[	1	]		25			±			1		[	1.1		]		24	±	1	[		1	]			10		±			1		[	0.9	]
1500						190	±			2	[	1	]		23			±			2		[	1		]		24	±	1	[		1	]			9		±			1		[	0.8	]
5000		T				196	±			4	[	1.1	]		26			±			1		[	1.2		]		26	±	2	[		1.1	]			11		±			2		[	1.1	]
Positive control						1528		±		135	[	8.3	]		258			±			20		[	11.6		]		192	±	32	[		7.9	]			183		±			2		[	17.2	]
Colonies/plate [factor] (Without S9 mix) ^a)^																																														
0						181	±			3					22			±			1							21	±	1							9		±			1				
50						180	±			2	[	1	]		23			±			2		[	1.1		]		21	±	1	[		1	]			9		±			1		[	1	]
150						187	±			3	[	1	]		25			±			1		[	1.1		]		25	±	1	[		1.2	]			8		±			2		[	0.9	]
500						185	±			5	[	1	]		23			±			1		[	1		]		25	±	3	[		1.2	]			8		±			2		[	0.9	]
1500						185	±			2	[	1	]		23			±			2		[	1.1		]		25	±	2	[		1.2	]			8		±			1		[	0.9	]
5000		T				188	±			4	[	1	]		26			±			1		[	1.2		]		26	±	1	[		1.2	]			11		±			1		[	1.2	]
Positive control						495	±			16	[	2.7	]		356			±			28		[	16.4		]		201	±	18	[		9.4	]			302		±			16		[	36.2	]

L-tryptophan fermentation product																																														
Conc.					Colonies/plate [factor] (With S9 mix) ^a)^																						Colonies/plate [factor] (Without S9 mix) ^a)^																			
(μg/plate)					*E. coli*																						*E. coli*																			
					WP2 *uvr*A																						WP2 *uvr*A																			
0					35									±	3												36								±	2										
0.15					37									±	2		[			1.1		]					33								±	2		[		0.9			]			
0.5					35									±	2		[			1		]					39								±	2		[		1.1			]			
1.5					35									±	2		[			1		]					34								±	1		[		0.9			]			
5					53									±	2		[			1.5		]					54								±	1		[		1.5			]			
15					81									±	1		[			2.3		]					77								±	1		[		2.1			]			
50					125									±	4		[			3.5		]					107								±	4		[		3			]			
100					153									±	4		[			4.3		]					134								±	3		[		3.7			]			
200					–									±	–		[			–		]					–								±	–		[		–			]			
Positive control					118									±	18		[			3.3		]					167								±	8		[		4.6			]			

L-tryptophan fermentation product																																														
Conc.					Colonies/plate [factor] (With S9 mix) ^a)^																						Colonies/plate [factor] (Without S9 mix) ^a)^																			
(μg/plate)					*S. typhimurium*																						*S. typhimurium*																			
					TA102																						TA102																			
0					329									±	29												325								±	21										
50					312									±	20				[	0.9		]					304								±	7		[		0.9			]			
150					325									±	22				[	1		]					344								±	23		[		1.1			]			
500					326									±	13				[	1		]					324								±	17		[		1			]			
1500					323									±	9				[	1		]					320								±	7		[		1			]			
5000	T				327									±	11				[	1		]					311								±	13		[		1			]			
Positive control					2045									±	360				[	6.2		]					1696								±	98		[		5.2			]			
Strain (With S9 mix)									Positive control							Dose (μg/plate)									Strain (Without S9 mix)							Positive control									Dose (μg/plate)					
TA 100									2-AA							1									TA 100							SA									0.5					
TA 1535									2-AA							2									TA1535							SA									0.5					
TA 98									B[a]P							1									TA98							2-NF									2					
TA 1537									2-AA							1									TA1537							ICR-191									0.5					
WP2 *uvr*A									2-AA							6									WP2 *uvr*A							4NQO									0.5					
TA 102									2-AA							6									TA 102							MMC									0.5					

a) Three plates/dose were used. No. of colonies of treated plate/No. of colonies of negative control plate Abbreviations: T, Turbidity in the treatment mixture; 2-AA, 2-aminoanthracene; SA, sodium azide; B[a]P, benzo[*a*]pyrene; ICR-191, acridine mutagen ICR 191; 4NQO, 4-nitroquinoline N-oxide; 2-NF, 2-Nitrofluorene; MMC, Mitomycin C.

### 3.2 *In vitro* mammalian chromosomal aberration test

As shown in Table 5, Table 6, Table 7, Table 8, Table 9, Table 10, cytotoxicity was observed at the highest concentrations of l-valine (24-S) and l-tryptophan (6-S and 24-S). There were no statistical significant increases in the frequencies of aberrant metaphases with structural (<5.0 % in both with gap (+gap) and without gap (-gap)) or numerical (almost 0 %) aberrations at any concentrations of the test compounds compared to the concurrent negative control. In addition, B[a]P, 4-NQO, MMC or CPA, induced a clear increase in the frequency of aberrant metaphases with structural aberrations as shown in Table 5, Table 6, Table 7, Table 8, Table 9, Table 10.

**Table 5:** Chromosomal aberrations induced in CHL cells treated with l-threonine fermentation product in the presence and absence of S9 mix.

l-threonine fermentation product											
Conc. (μg/mL)	No. of cells examined	6 -h treatment (+ S9 mix)									
		Aberrations						PP + ER	No. of aberrant metaphases		RICC (%)
		Chromosome type		Chromatid type		Others	Gaps	No.	+Gaps	-Gaps	
		csb	cse	ctb	cte				No.	No.	
0	150	0	0	0	0	0	0	0	0	0	100
	150	0	0	0	1	0	0	0	1	1	
	300	(0.0)	(0.0)	(0.0)	(0.3)	(0.0)	0.0)				
								(0.00 %)	(0.33 %)	(0.33 %)	
500	150	0	0	0	0	0	0	0	0	0	94
	150	0	0	0	0	0	0	0	0	0	
	300	(0.0)	(0.0)	(0.0)	(0.0)	(0.0)	(0.0)				
								(0.00 %)	(0.00 %)	(0.00 %)	
1000	150	0	0	0	0	0	0	0	0	0	98
	150	0	0	0	0	0	0	0	0	0	
	300	(0.0)	(0.0)	(0.0)	(0.0)	(0.0)	(0.0)				
								(0.00 %)	(0.00 %)	(0.00 %)	
2000	150	0	0	0	1	0	0	0	1	1	87
	150	0	0	0	0	0	0	0	0	0	
	300	(0.0)	(0.0)	(0.0)	(0.3)	(0.0)	(0.0)				
								(0.00 %)	(0.33 %)	(0.33 %)	
CPA 5	150	0	1	3	31	0	0	0	35	35	59
	150	1	0	1	30	0	0	0	32	32	
	300	(0.3)	(0.3)	(1.3)	(20.3)	(0.0)	(0.0)				
								(0.00 %)	(22.33 %)	(22.33 %)	

Conc. (μg/mL)	No. of cells examined	6 -h treatment (- S9 mix)									
		Aberrations						PP + ER	No. of aberrant metaphases		RICC (%)
		Chromosome type		Chromatid type		Others	Gaps	No.	+Gaps	-Gaps	
		csb	cse	ctb	cte				No.	No.	
0	150	0	0	0	0	0	0	0	0	0	100
	150	0	0	0	0	0	0	0	0	0	
	300	(0.0)	(0.0)	(0.0)	(0.0)	(0.0)	(0.0)				
								(0.00 %)	(0.00 %)	(0.00 %)	
500	150	0	0	0	0	0	0	0	0	0	91
	150	0	0	0	0	0	0	0	0	0	
	300	(0.0)	(0.0)	(0.0)	(0.0)	(0.0)	(0.0)				
								(0.00 %)	(0.00 %)	(0.00 %)	
1000	150	0	0	0	0	0	0	0	0	0	87
	150	0	0	0	0	0	0	0	0	0	
	300	(0.0)	(0.0)	(0.0)	(0.0)	(0.0)	(0.0)				
								(0.00 %)	(0.00 %)	(0.00 %)	
2000	150	0	0	0	0	0	0	0	0	0	73
	150	0	0	0	0	0	1	0	1	0	
	300	(0.0)	(0.0)	(0.0)	(0.0)	(0.0)	(0.3)				
								(0.00 %)	(0.33 %)	(0.00 %)	
MMC 0.1	150	2	0	1	31	0	0	0	33	33	27
	150	1	0	2	27	0	1	0	31	30	
	300	(1.0)	(0.0)	(1.0)	(19.3)	(0.0)	(0.3)				
								(0.00 %)	(21.33 %)	(21.00 %)	

Abbreviations: ctb break of chromatid; cte exchange of chromatid; csb break of chromosome; cse exchange of chromosome; Gaps Chromosome type + Chromatid type gaps; Other Metaphases with more than 10 aberrations (including gaps) or with chromosome fragmentation; PP polyploidy; ER endoreduplication; CPA Cyclophosphamide monohydrate MMC, Mitomycin C.

**Table 6:** Chromosomal aberrations induced in CHL cells treated with l-threonine fermentation product in the absence of S9 mix.

L-threonine fermentation product											
Conc. (μg/mL)	No. of cells examined	24 -h treatment (- S9 mix)									
								PP + ER	No. of aberrant metaphases		RICC (%)
		Chromosome type		Chromatid type		Others	Gaps	No.	+Gaps	-Gaps	
		csb	cse	ctb	cte				No.	No.	
0	150	0	0	0	0	0	0	0	0	0	100
	150	0	0	0	0	0	0	0	0	0	
	300	(0.0)	(0.0)	(0.0)	(0.0)	(0.0)	(0.0)				
								(0.00 %)	(0.00 %)	(0.00 %)	
500	150	0	0	0	0	0	1	0	1	0	92
	150	0	0	0	0	0	1	0	1	0	
	300	(0.0)	(0.0)	(0.0)	(0.0)	(0.0)	(0.7)				
								(0.00 %)	(0.67 %)	(0.00 %)	
1000	150	0	0	0	0	0	1	0	1	0	101
	150	0	0	0	0	0	0	0	0	0	
	300	(0.0)	(0.0)	(0.0)	(0.0)	(0.0)	(0.3)				
								(0.00 %)	(0.33 %)	(0.00 %)	
2000	150	0	0	0	0	0	0	0	0	0	93
	150	0	0	0	0	0	0	0	0	0	
	300	(0.0)	(0.0)	(0.0)	(0.0)	(0.0)	(0.0)				
								(0.00 %)	(0.00 %)	(0.00 %)	
MMC 0.05	150	1	1	2	29	0	0	0	33	33	30
	150	0	0	1	31	0	1	0	33	32	
	300	(0.3)	(0.3)	(1.0)	(20.0)	(0.0)	(0.3)				
								(0.00 %)	(22.00 %)	(21.67 %)	

Abbreviations: ctb, break of chromatid; cte, exchange of chromatid; csb, break of chromosome; cse, exchange of chromosome; Gaps, Chromosome type + Chromatid type gaps; Other, Metaphases with more than 10 aberrations (including gaps) or with chromosome fragmentation; PP, polyploidy; ER, endoreduplication; MMC, Mitomycin C.

**Table 7:** Chromosomal aberrations induced in CHL cells treated with l-valine fermentation product in the presence and absence of S9 mix.

L-valine fermentation product											
Conc. (μg/mL)	No. of cells examined	6 -h treatment (+ S9 mix)									
		Aberrations						PP + ER	No. of aberrant metaphases		RICC (%)
		Chromosome type		Chromatid type		Others	Gaps	No.	+Gaps	-Gaps	
		csb	cse	ctb	cte				No.	No.	
0	150	0	0	0	1	0	1	0	2	1	100
	150	0	0	0	0	0	1	0	1	0	
	(mean)	(0.0)	(0.0)	(0.0)	(0.5)	(0.0)	(1.0)	(0.0)	(1.5)	(0.5)	
								(0.00 %)	(1.00 %)	(0.33 %)	
250	150	0	0	0	3	0	1	0	3	2	98
	150	0	0	0	0	0	0	0	0	0	
	(mean)	(0.0)	(0.0)	(0.0)	(1.5)	(0.0)	(0.5)	(0.0)	(1.5)	(1.0)	
								(0.00 %)	(1.00 %)	(0.67 %)	
500	150	0	0	0	1	0	0	0	1	1	104
	150	0	0	0	0	0	1	0	1	0	
	(mean)	(0.0)	(0.0)	(0.0)	(0.5)	(0.0)	(0.5)	(0.0)	(1.0)	(0.5)	
								(0.00 %)	(0.67 %)	(0.33 %)	
1000^T^	150	0	0	0	0	0	2	0	2	0	82
	150	0	0	0	0	0	1	0	1	0	
	(mean)	(0.0)	(0.0)	(0.0)	(0.0)	(0.0)	(1.5)	(0.0)	(1.5)	(0.0)	
								(0.00 %)	(1.00 %)	(0.00 %)	
B[a]P 20	150	0	0	2	58	0	6	0	45	42	58
	150	1	1	5	54	0	3	0	43	41	
	(mean)	(0.5)	(0.5)	(3.5)	(56.0)	(0.0)	(4.5)	(0.0)	(44.0)	(41.5)	
								(0.00 %)	(29.33 %)	(27.67 %)**	

Conc. (μg/mL)	No. of cells examined	6 -h treatment (- S9 mix)									
		Aberrations						PP + ER	No. of aberrant metaphases		RICC (%)
		Chromosome type		Chromatid type		Others	Gaps	No.	+Gaps	-Gaps	
		csb	cse	ctb	cte				No.	No.	
0	150	0	0	0	1	0	0	0	1	1	100
	150	0	0	0	1	0	3	0	4	1	
	(mean)	(0.0)	(0.0)	(0.0)	(1.0)	(0.0)	(1.5)	(0.0)	(2.5)	(1.0)	
								(0.00 %)	(1.67 %)	(0.67 %)	
250	150	0	0	0	0	0	0	0	0	0	96
	150	0	0	0	2	0	0	0	2	2	
	(mean)	(0.0)	(0.0)	(0.0)	(1.0)	(0.0)	(0.0)	(0.0)	(1.0)	(1.0)	
								(0.00 %)	(0.67 %)	(0.67 %)	
500	150	1	0	0	0	0	1	0	2	1	90
	150	0	0	0	0	0	1	0	1	0	
	(mean)	(0.5)	(0.0)	(0.0)	(0.0)	(0.0)	(1.0)	(0.0)	(1.5)	(0.5)	
								(0.00 %)	(1.00 %)	(0.33 %)	
1000^T^	150	0	0	0	1	0	2	0	3	1	71
	150	0	0	1	0	0	0	1	1	1	
	(mean)	(0.0)	(0.0)	(0.5)	(0.5)	(0.0)	(1.0)	(0.5)	(2.0)	(1.0)	
								(0.33 %)	(1.33 %)	(0.67 %)	
B[a]P 20	150	0	0	0	65	0	0	0	33	33	56
	150	0	0	9	48	0	2	1	23	22	
	(mean)	(0.0)	(0.0)	(4.5)	(56.5)	(0.0)	(1.0)	(0.5)	(28.0)	(27.5)	
								(0.33 %)	(18.67 %)	(18.33 %)**	

** Significantly different from the negative control at *P*<0.01 (Fisher’s exact test).

Abbreviations: T, Turbid at the end of the treatment; ctb, break of chromatid; cte, exchange of chromatid; csb, break of chromosome; cse, exchange of chromosome; Gaps, Chromosome type + Chromatid type gaps; Other, Metaphases with more than 10 aberrations (including gaps) or with chromosome fragmentation; PP, polyploidy; ER, endoreduplication; B[a]P.benzo[*a*]pyrene.

**Table 8:** Chromosomal aberrations induced in CHL cells treated with l-valine fermentation product in the absence of S9 mix.

L-valine fermentation product											
Conc. (μg/mL)	No. of cells examined	24 -h treatment (- S9 mix)									
		Aberrations						PP + ER	No. of aberrant metaphases		RICC (%)
		Chromosome type		Chromatid type		Others	Gaps	No.	+Gaps	-Gaps	
		csb	cse	ctb	cte				No.	No.	
0	150	0	0	0	2	0	0	0	1	1	100
	150	0	0	0	0	0	0	0	0	0	
	(mean)	(0.0)	(0.0)	(0.0)	(1.0)	(0.0)	(0.0)	(0.0)	(0.5)	(0.5)	
								(0.00 %)	(0.33 %)	(0.33 %)	
250	150	0	0	1	0	0	1	0	2	1	90
	150	0	0	0	1	0	0	0	1	1	
	(mean)	(0.0)	(0.0)	(0.5)	(0.5)	(0.0)	(0.5)	(0.0)	(1.5)	(1.0)	
								(0.00 %)	(1.00 %)	(0.67 %)	
500	150	0	0	0	0	0	0	0	0	0	73
	150	0	0	0	0	0	0	0	0	0	
	(mean)	(0.0)	(0.0)	(0.0)	(0.0)	(0.0)	(0.0)	(0.0)	(0.0)	(0.0)	
								(0.00 %)	(0.00 %)	(0.00 %)	
800	150	0	0	0	0	0	0	0	0	0	56
	150	0	0	0	0	0	0	0	0	0	
	(mean)	(0.0)	(0.0)	(0.0)	(0.0)	(0.0)	(0.0)	(0.0)	(0.0)	(0.0)	
								(0.00 %)	(0.00 %)	(0.00 %)	
1000^T^	150	0	0	1	1	0	0	0	2	2	41
	150	0	0	0	0	0	0	1	0	0	
	(mean)	(0.0)	(0.0)	(0.5)	(0.5)	(0.0)	(0.0)	(0.5)	(1.0)	(1.0)	
								(0.33 %)	(0.67 %)	(0.67 %)	
4NQO 0.4	150	1	0	10	45	0	1	0	26	26	56
	150	0	0	8	56	0	4	2	32	31	
	(mean)	(0.5)	(0.0)	(9.0)	(50.0)	(0.0)	(2.5)	(1.0)	(29.0)	(28.5)	
								(0.67 %)	(19.33 %)	(19.00 %)**	

** Significantly different from the negative control at *P*<0.01 (Fisher’s exact test).

Abbreviations: T, Turbid at the end of the treatment; ctb, break of chromatid; cte, exchange of chromatid; csb, break of chromosome; cse, exchange of chromosome; Gaps, Chromosome type + Chromatid type gaps; Other, Metaphases with more than 10 aberrations (including gaps) or with chromosome fragmentation; PP, polyploidy; ER, endoreduplication; 4NQO, 4-nitroquinoline N-oxide.

**Table 9:** Chromosomal aberrations induced in CHL cells treated with l-tryptophan fermentation product in the presence and absence of S9 mix.

L-tryptophan fermentation product											
Conc. (μg/mL)	No. of cells examined	6 -h treatment (+ S9 mix)									
		Aberrations						PP + ER	No. of aberrant metaphases		RICC (%)
		Chromosome type		Chromatid type		Others	Gaps	No.	+Gaps	-Gaps	
		csb	cse	ctb	cte				No.	No.	
0	150	0	0	0	1	0	1	0	2	1	100
	150	0	0	0	1	0	1	0	2	1	
	(mean)	(0.0)	(0.0)	(0.0)	(1.0)	(0.0)	(1.0)	(0.0)	(2.0)	(1.0)	
								(0.00 %)	(1.33 %)	(0.67 %)	
125	150	0	0	0	0	0	0	0	0	0	92
	150	0	0	0	2	0	0	0	2	2	
	(mean)	(0.0)	(0.0)	(0.0)	(1.0)	(0.0)	(0.0)	(0.0)	(1.0)	(1.0)	
								(0.00 %)	(0.67 %)	(0.67 %)	
250	150	0	0	0	0	0	0	0	0	0	100
	150	0	0	0	0	0	0	0	0	0	
	(mean)	(0.0)	(0.0)	(0.0)	(0.0)	(0.0)	(0.0)	(0.0)	(0.0)	(0.0)	
								(0.00 %)	(0.00 %)	(0.00 %)	
500^T^	150	0	0	0	0	0	1	0	1	0	94
	150	0	0	0	0	0	0	0	0	0	
	(mean)	(0.0)	(0.0)	(0.0)	(0.0)	(0.0)	(0.5)	(0.0)	(0.5)	(0.0)	
								(0.00 %)	(0.33 %)	(0.00 %)	
B[a]P 20	150	1	0	4	53	0	1	0	36	35	58
	150	0	1	5	62	0	3	0	52	49	
	(mean)	(0.5)	(0.5)	(4.5)	(57.5)	(0.0)	(2.0)	(0.0)	(44.0)	(42.0)	
								(0.00 %)	(29.33 %)	(28.00 %)**	

Conc. (μg/mL)	No. of cells examined	6 -h treatment (- S9 mix)									
		Aberrations						PP + ER	No. of aberrant metaphases		RICC (%)
		Chromosome type		Chromatid type		Others	Gaps	No.	+Gaps	-Gaps	
		csb	cse	ctb	cte				No.	No.	
0	150	0	0	0	0	0	2	0	2	0	100
	150	0	0	0	0	0	0	0	0	0	
	(mean)	(0.0)	(0.0)	(0.0)	(0.0)	(0.0)	(1.0)	(0.0)	(1.0)	(0.0)	
								(0.00 %)	(0.67 %)	(0.00 %)	
125	150	0	0	0	0	0	0	0	0	0	97
	150	0	0	0	0	0	0	0	0	0	
	(mean)	(0.0)	(0.0)	(0.0)	(0.0)	(0.0)	(0.0)	(0.0)	(0.0)	(0.0)	
								(0.00 %)	(0.00 %)	(0.00 %)	
250	150	0	0	0	1	0	0	0	1	1	77
	150	0	0	0	0	0	0	0	0	0	
	(mean)	(0.0)	(0.0)	(0.0)	(0.5)	(0.0)	(0.0)	(0.0)	(0.5)	(0.5)	
								(0.00 %)	(0.33 %)	(0.33 %)	
500^T^	150	0	0	0	0	0	0	0	0	0	46
	150	0	0	0	0	0	0	1	0	0	
	(mean)	(0.0)	(0.0)	(0.0)	(0.0)	(0.0)	(0.0)	(0.5)	(0.0)	(0.0)	
								(0.33 %)	(0.00 %)	(0.00 %)	
4NQO 0.4	150	0	0	3	68	1	0	0	23	23	65
	150	0	0	4	31	0	1	0	17	16	
	(mean)	(0.0)	(0.0)	(3.5)	(49.5)	(0.5)	(0.5)	(0.0)	(20.0)	(19.5)	
								(0.00 %)	(13.33 %)	(13.00 %)**	

** Significantly different from the negative control at P<0.01 (Fisher’s exact test).

Abbreviations: T, Turbid at the end of the treatment; ctb, break of chromatid; cte, exchange of chromatid; csb, break of chromosome; cse, exchange of chromosome; Gaps, Chromosome type + Chromatid type gaps; Other, Metaphases with more than 10 aberrations (including gaps) or with chromosome fragmentation; PP, polyploidy; ER, endoreduplication; B[a]P, benzo[*a*]pyrene; 4NQO, 4-nitroquinoline N-oxide.

**Table 10:** Chromosomal aberrations induced in CHL cells treated with l-tryptophan fermentation product in the absence of S9 mix.

L-tryptophan fermentation product											
Conc. (μg/mL)	No. of cells examined	24 -h treatment (- S9 mix)									
		Aberrations						PP + ER	No. of aberrant metaphases		RICC (%)
		Chromosome type		Chromatid type		Others	Gaps	No.	+Gaps	-Gaps	
		csb	cse	ctb	cte				No.	No.	
0	150	0	0	0	0	0	1	0	1	0	100
	150	0	0	0	0	0	0	0	0	0	
	(mean)	(0.0)	(0.0)	(0.0)	(0.0)	(0.0)	(0.5)	(0.0)	(0.5)	(0.0)	
								(0.00 %)	(0.33 %)	(0.00 %)	
87.5	150	0	0	0	0	0	0	0	0	0	92
	150	0	0	0	0	0	0	0	0	0	
	(mean)	(0.0)	(0.0)	(0.0)	(0.0)	(0.0)	(0.0)	(0.0)	(0.0)	(0.0)	
								(0.00 %)	(0.00 %)	(0.00 %)	
175	150	0	0	0	0	0	0	0	0	0	66
	150	0	0	0	0	0	0	0	0	0	
	(mean)	(0.0)	(0.0)	(0.0)	(0.0)	(0.0)	(0.0)	(0.0)	(0.0)	(0.0)	
								(0.00 %)	(0.00 %)	(0.00 %)	
310	150	0	0	0	2	0	0	0	2	2	54
	150	0	0	0	0	0	0	0	0	0	
	(mean)	(0.0)	(0.0)	(0.0)	(1.0)	(0.0)	(0.0)	(0.0)	(1.0)	(1.0)	
								(0.00 %)	(0.67 %)	(0.67 %)	
350	150	0	0	0	1	0	0	0	1	1	42
	150	0	0	0	0	0	0	0	0	0	
	(mean)	(0.0)	(0.0)	(0.0)	(0.5)	(0.0)	(0.0)	(0.0)	(0.5)	(0.5)	
								(0.00 %)	(0.33 %)	(0.33 %)	
4NQO 0.4	150	1	0	7	65	1	0	0	29	29	61
	150	1	0	6	33	3	1	0	23	22	
	(mean)	(1.0)	(0.0)	(6.5)	(49.0)	(2.0)	(0.5)	(0.0)	(26.0)	(25.5)	
								(0.00 %)	(17.33 %)	(17.00 %)**	

** Significantly different from the negative control at P<0.01 (Fisher’s exact test).

Abbreviations: ctb, break of chromatid; cte, exchange of chromatid; csb, break of chromosome; cse, exchange of chromosome; Gaps, Chromosome type + Chromatid type gaps; Other, Metaphases with more than 10 aberrations (including gaps) or with chromosome fragmentation; PP, polyploidy; ER, endoreduplication; 4NQO, 4-nitroquinoline N-oxide.

### 3.3 Acute oral toxicity

Under the present laboratory conditions, there were no mortalities, no clinical signs, no changes of body weight, and no macroscopic findings in necropsy at the dose of 300 mg/kg b.w. and 2000 mg/kg b.w. as shown in Table 11, Table 12, Table 13. Based on the results of this study, when each test compound was dosed to rats by a fixed dose procedure method, all test compounds were categorized as GHS category 5/unclassified.

**Table 11:** Body weights of the three amino acids fermentation product-treated animals.

L-threonine fermentation product	DAYS	GROUPS (mg/kg)		
		G1 (300)	G2 (2000)	G3 (2000)
	1	165	154	175.75 ± 7.46
	8	185	170	189.25 ± 0.96
	15	198	189	205.50 ± 7.72
	N	1	1	4
L-valine fermentation product	DAYS	GROUPS (mg/kg)		
		G1 (300)	G2 (2000)	G3 (2000)
	1	176.02	181.45	184.18 ± 2.70
	2	192.18	200.42	207.22 ± 2.52
	4	201.49	210.52	214.19 ± 2.99
	8	217.67	222.11	223.27 ± 3.75
	15	238.23	231.95	238.13 ± 6.78
	N	1	1	4
L-tryptophan fermentation product	DAYS	GROUPS (mg/kg)		
		G1 (300)	G2 (2000)	G3 (2000)
	1	200.46	211.93	204.58 ± 5.27
	2	225.25	232.57	222.22 ± 6.34
	4	239.93	243.38	233.87 ± 8.28
	8	255.59	257.74	246.69 ± 11.53
	15	280.65	289.47	269.91 ± 11.76
	N	1	1	4

The day of administration was designated as Day 1.

Data are expressed as mean ± S.D.

**Table 12:** Necropsy findings of the three amino acids fermentation product-treated animals.

NECROPSY FINDINGS					FEMALE
Test compound	ORGANS	FINDINGS	GROUPS (mg/kg)		
			G1 (300)	G2 (2000)	G3 (2000)
L-threonine fermentation product	No gross findings		1	1	4
	N		1	1	4
L-valine fermentation product	No gross findings		1	1	4
	N		1	1	4
L-tryptophan fermentation product	No gross findings		1	1	4
	N		1	1	4

**Table 13:** Clinical signs of the three amino acids fermentation product treated animals.

CLINICAL SIGNS						FEMALE
Test compound	DAYS	SIGNS	GROUPS (mg/kg)			
			G1 (300)	G2 (2000)	G3 (2000)	
L-threonine fermentation product	1-14	Normal	1 / 1	1 / 1	4 / 4	
	15	Normal	1 / 1	1 / 1	4 / 4	
L-valine fermentation product	1-14	Normal	1 / 1	1 / 1	4 / 4	
	15	Normal	1 / 1	1 / 1	4 / 4	
L-tryptophan fermentation product	1-14	Normal	1 / 1	1 / 1	4 / 4	
	15	Normal	1 / 1	1 / 1	4 / 4	

The day of administration was designated as Day 1.

Number of animals with the sign/Number of animals examined.

## 4 Discussion

The present study was performed to evaluate the toxicity of the three amino acid additives for *in vitro* genotoxicity and *in vivo* acute animal toxicity under the OECD Guidelines and the GLP regulations for ingredient or other biological uses.

To evaluate potential genotoxicity of the three amino acids fermentation products, bacterial reverse mutation tests and chromosomal aberration tests were carried out. In the Ames assay investigating the potential of the three amino acid additives to induce gene mutations at concentrations of up to 3000 μg/plate (l-threonine) and 5000 μg/plate (l-valine and l-tryptophan fermentation products), no biologically-relevant increase in revertant colony numbers was found in any of the tested strains *S. typhimurium* TA100, TA1535, TA98, TA1537 and in combination with *E. coli* WP2 uvrA in the presence or absence of metabolic activation system. Also, a mammalian cytogenetic assay in cultured CHL cells for the direct observation in identifying chromosomal aberrations and no statistically-significant or concentration-dependent increase was found in the frequencies of aberrant metaphases with structural or numerical aberrations at all concentrations of the test compound compared to the concurrent negative control. Interestingly, a significant increase of the revertants per plate was found in WP2 uvrA with treatment of l-tryptophan fermentation product. It was considered that the addition of l-tryptophan fermentation product (Table 1) could lead to false positives in WP2 uvrA, resulting in the increases of revertants per plate [27]. It was considered that increases in numbers of colonies in WP2 uvrA may have been due to the content of l-tryptophan (63.09% in total) as shown in Table 1. To double-check the increase of the revertants in WP2 uvrA by l-tryptophan fermentation product, *S. typhimurium* TA102, one of recommended strains in the OECD 471 Guideline, was tested at concentration levels (50, 150, 500, 1500, and 5000 μg/plate) of l-tryptophan fermentation product. As shown in Table 4, there was no increase in the number of revertants compared to the negative control in the TA102 strain. However, the positive control as 2-aminoanthracene showed a 6-fold increase of revertants compared to the negative control in the same strain. Therefore, the l-tryptophan fermentation product is non-mutagenic under the conditions of the present study. In conclusion, the three amino acid additives are non-mutagenic under the laboratory conditions of the present study.

In this acute oral toxicity study, two separate independent GLP-certified research institutes, one in the Republic of Korea and the other in the UK. Each independently conducted the same acute oral toxicity tests at the sponsor’s request. The animals were sourced from two different suppliers. The results of the acute oral toxicity test demonstrated that the three amino acid additives categorized as GHS category 5/unclassified. No mortalities, clinical signs, change of body weight, and gross abnormal necropsy findings were observed as results of the three amino acid additives at the dose of 2000 mg/kg in both Sprague-Dawley in Chemon in the Republic of Korea and Wistar rats in Envigo in the UK. These results suggest that the three amino acid additives did not demonstrate toxicity regardless of the animal strains being sourced from two different outbred rodent suppliers. In this study, the three amino acid additives at dose of 2000 mg/kg had no adverse effect on the tested rats through Day 1–14 in clinical observations, changes of body weights, and necropsy findings. Therefore, this study indicates that the three amino acid additives do not cause acute toxicity effects at the doses tested and the LD_50_ value was >2000 mg/kg. The determination of LD_50_ from acute toxicity studies is usually the initial step to serve as the basis for classification and provides initial information on the mode of toxic action of a test compound. According to the chemical labeling and classification of acute systemic toxicity recommended by the OECD, the three amino acid additives were assigned as class 5/unclassified at which dose level the animals are expected to survive under the present laboratory conditions. Furthermore, the data of the acute toxicity study should be further analyzed to increase the confidence in establishing the compounds safety as ingredients or for other biological uses. However, further evaluative toxicity studies should be performed to investigate toxicological profiles such as the target organ or delayed toxicity for the three amino acid additives for up to 90 days in rats, although there were no toxicological effects found in clinical signs, changes of body weights, and necropsy findings under the present laboratory conditions in the acute toxicity study.

In addition, l-threonine, l-valine and l-tryptophan on the list of European Food Safety Authority (EFSA) produced by an aerobic fermentation process using *C. glutamicum*, are generally recognized as safe (GRAS) for humans and food producing animals and testing showed that there is no exposure risk to humans consuming tissues or products from the target animals [[28], [29], [30]]. As shown in Table 1, toxicological changes in gene mutations, chromosome aberration and acute oral toxicity were evaluated in the presence of other unknown impurities and derivatives from *C. glutamicum* in amino acid additives. Under the present laboratory conditions, *C. glutamicum* was found to be non-mutagenic, non-clastogenic in genotoxicity, and non-toxic in the acute oral toxicity test.

## 5 Conclusion

The present study and the results presented herein, support the safety of the three amino acid additives with *C. glutamicum* in terms of genotoxicity and acute oral toxicity in accordance with the OECD guidelines and the principles of GLP. For the first time, these results show that the three amino acid additives with *C. glutamicum* are safe with no adverse effects and may be applied as an ingredient in products for food-producing animals or other biological uses.

## CRediT authorship contribution statement

**Ki-Young Kang:** Conceptualization, Writing - original draft, Writing - review & editing, Project administration. **Min-Sub Kim:** Conceptualization, Methodology, Writing - original draft, Writing - review & editing, Project administration. **Min-Seung Lee:** Methodology. **Jeong-Ja Oh:** Formal analysis, Writing - review & editing. **Seulgi An:** Resources, Writing - review & editing. **Dhanbee Park:** Resources, Writing - review & editing. **In Kyoung Heo:** Writing - review & editing. **Hyun-Kul Lee:** Resources, Writing - review & editing. **Si-Whan Song:** Writing - review & editing. **Sun-Don Kim:** Methodology, Formal analysis, Writing - original draft, Writing - review & editing, Supervision, Project administration.

## Declaration of Competing Interest

The authors declare that there is no conflict of interest.
